# Rare Presentation of Incarcerated Incisional Richter's Hernia of the Cecum

**DOI:** 10.7759/cureus.16971

**Published:** 2021-08-07

**Authors:** Gabriella Schmuter, Nisha Narula, Indraneil Mukherjee

**Affiliations:** 1 Surgery, Northwell Health Staten Island University Hospital, Staten Island, USA

**Keywords:** hernia, richter's hernia, surgery, incarceration, general surgery, partial enterocele

## Abstract

Richter’s hernia, also called a partial enterocele, involves a protrusion of peritoneum with subsequent strangulation or incarceration of only part of the lumen of the anti-mesenteric portion of the small bowel through a fascial defect. We report a rare presentation of incarcerated incisional Richter’s hernia of the cecum in a 39-year-old female. The patient presented with acute abdominal pain that gradually improved. Physical examination revealed right lower quadrant tenderness and nodularity just above an abdominoplasty scar. Subsequent computed tomography scan demonstrated a 1 cm by 1 cm hypovascular pocket arising from the cecum with protrusion into the anterior abdominal wall. The hernia was successfully repaired surgically with resolution of symptoms. It is essential for clinicians to be mindful of the diagnosis of Richter’s hernia on the differential for abdominal pain as the risk of detrimental outcomes increases with delayed surgical intervention.

## Introduction

Richter’s hernia involves a protrusion of peritoneum with subsequent strangulation or incarceration of only part of the lumen of the anti-mesenteric portion of the small bowel through a fascial defect. As a result, the lumen is incompletely contained in the defect. Timely repair of a Richter’s hernia is instrumental as mortality increases with delays in surgical intervention due to the high risk of strangulation and necrosis. The insidious nature and late presentation of a Richter’s hernia, as well as its relative rarity, may lead to late diagnosis or misdiagnosis [1,2].

## Case presentation

A 39-year-old female patient initially presented to the emergency department after a fall at home. Computed tomography (CT) imaging of the abdomen and pelvis at the emergency department did not reveal any major pathology. She visited the clinic a month later for persistent tenderness at the right lower quadrant of the abdomen. She denied any exacerbating or relieving factors. The patient additionally denied any associated symptoms, including fever, chest pain, shortness of breath, palpitations, dysuria, or known exposure to coronavirus disease 2019 (COVID-19). The patient has a past surgical history of an appendectomy, three cesarean sections with bilateral salpingo-oophorectomy, and abdominoplasty. Her family history was only remarkable for unspecified heart disease.

**Figure 1:**
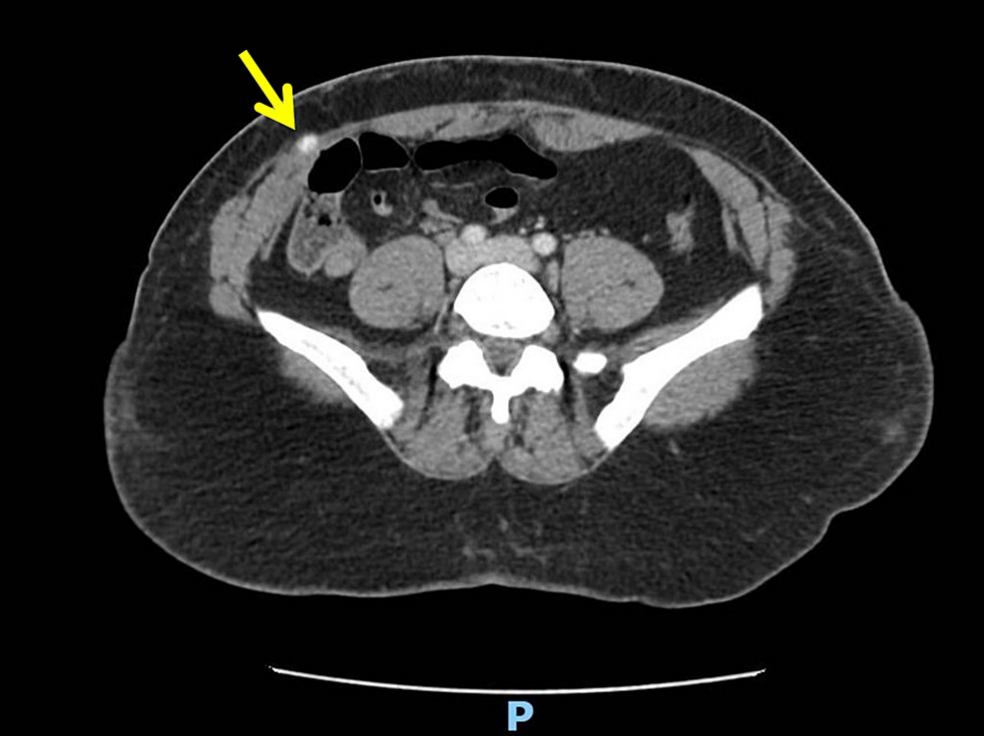
CT of abdomen in emergency department CT image of abdomen taken when the patient came to the emergency department for an unrelated fall. Upon close inspection, an area of hypervascularity can be appreciated near the anterior abdominal wall without protrusion.

At the clinic, physical examination revealed right lower quadrant tenderness and nodularity 2 cm above her abdominoplasty scar that was potentially overlapping the site of a previous appendectomy incision. The nodularity could not be easily reduced into the abdominal cavity. On closer review of the previous CT scan from her emergency room visit, there was a newly appreciated area of hypervascularity near the palpable nodularity (Figure 1).

**Figure 2:**
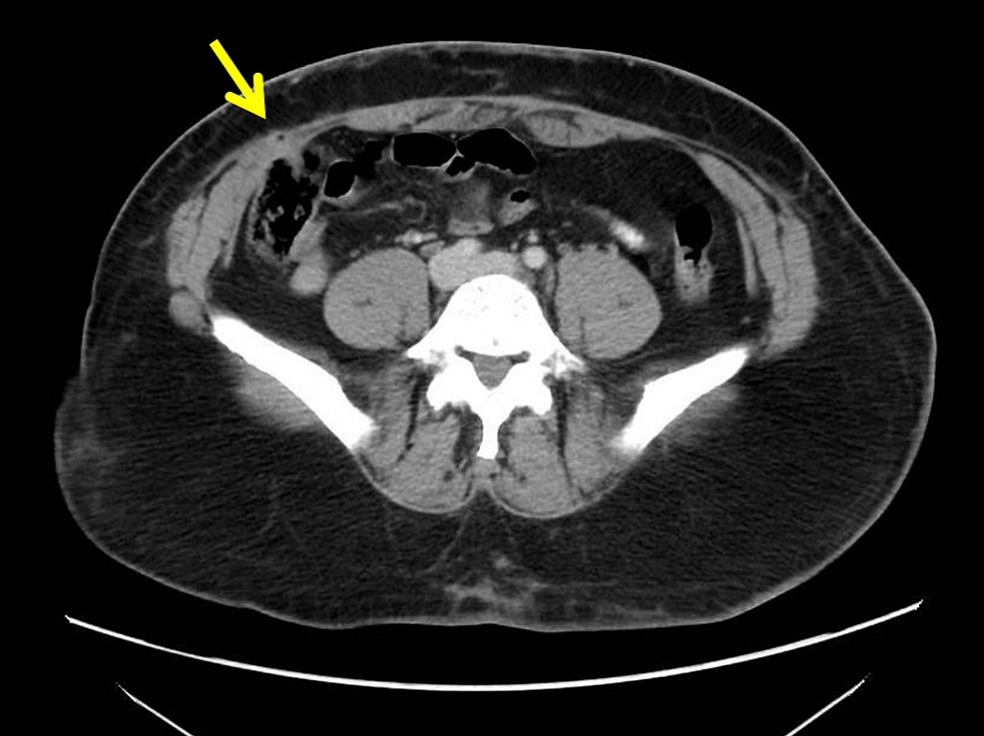
Preoperative CT of abdomen CT image of abdomen preoperatively demonstrating a 1 cm by 1 cm outpouching of hypervascularity through the anterior abdominal wall. This abdominal pathology was not appreciated in the previous CT from the emergency department months prior.

Given the likelihood of a hematoma or a fascial defect at the site of the incision along with the concern that this suspected hernia was not reducible, urgent imaging was warranted to rule out incarceration or strangulation. Subsequent preoperative CT imaging revealed a 1 cm by 1 cm hypovascular pocket arising from the cecum and protruding into the anterior abdominal wall without any inflammatory changes (Figure 2). Such findings confirmed the initial suspicion of a fascial defect as the cause of the nodularity, and strongly suggested incarceration of a cecal hernia.

**Figure 3:**
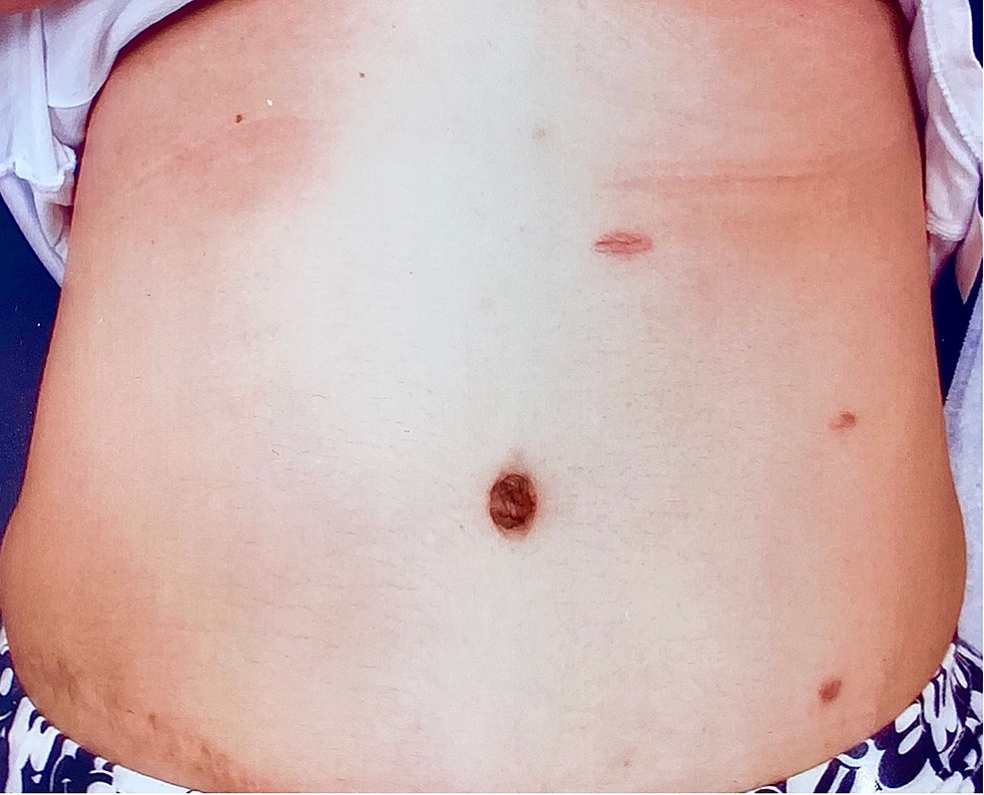
Postoperative photograph of abdomen Photograph of patient’s abdomen postoperatively demonstrating three small, well-healed incisions. A scar in the right lower quadrant from a previous open appendectomy is noted.

Shortly after, the patient appropriately underwent an exploratory laparoscopy. The abdomen was entered in the left upper quadrant and left lower quadrant with three optical trocars (Figure 3). It was appreciated that both omentum and cecum were adhered to the fascial defect of her previous appendectomy site. Lysis of adhesions was completed with scissors, and extensive dissection revealed an incarcerated Richter’s hernia of the cecum. The hernia was non-obstructing as there was only a knuckle of cecum herniating into a 1 cm defect. There was no evidence of bowel ischemia. We were unable to reduce the hernia because of dense adhesions. After upsizing a trocar site, an endovascular stapler was used to transect the cecum tangentially. This allowed us to pull on and dissect the herniated portion of the cecum. This component was sent as a specimen to the pathology laboratory. Incisional hernia repair was then performed in a primary fashion due to the small defect size and contaminated nature of the case. The fascia was closed with two layers of running #2 PDS (polydioxanone suture) Quill sutures (Westwood, MA: Surgical Specialties Corporation).

**Figure 4:**
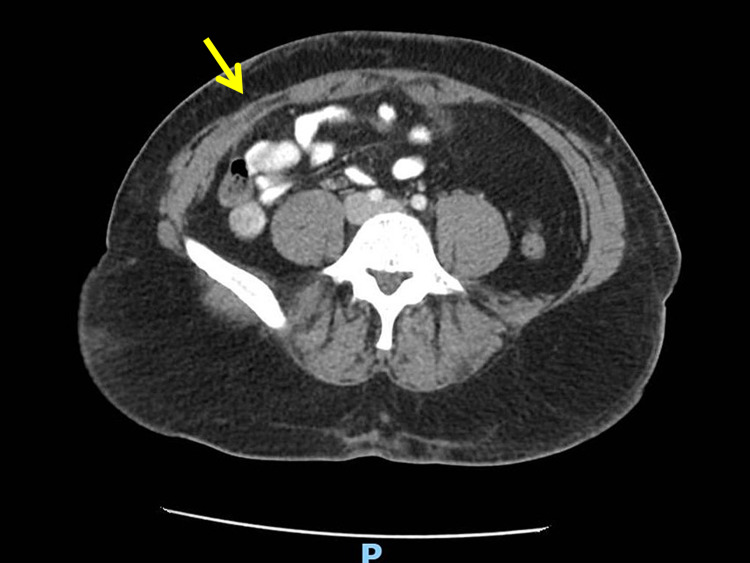
Postoperative CT of abdomen CT image of abdomen postoperatively revealing full resolution of fascial defect. No abdominal pathology is appreciated at this visit.

The patient did well postoperatively and was discharged home from the recovery unit. The pathology laboratory reported the patient’s specimen as demonstrating benign disease with nonspecific chronic inflammation. A repeat CT of the abdomen and pelvis demonstrated full resolution of the incisional Richter’s hernia with lack of pathology at the surgical sites (Figure 4).

## Discussion

The initial differential for abdominal pain is very broad, and thus a careful attention to patient’s history, particularly those of symptoms as well as prior operations, physical examination, laboratory studies, and imaging is required. This tailors the differential based on the likelihood of diagnosis in that patient [3].

It is important to note that the patient’s physical findings of tenderness and nodularity at the right lower quadrant was near the same site as a prior appendectomy, even though her cutaneous scar was excised during the subsequent abdominoplasty. This context is highly suggestive of herniation given the inherent weakness to the fascia from a past incision. A diagnosis of hematoma would have been sufficient if there was no history of surgery at the site.

Additionally, the inability to reduce the mass was highly suggestive of incarceration or strangulation. At this stage, CT imaging was able to demonstrate the likely diagnosis of a hernia given the protrusion of the cecum through the anterior abdominal wall. It is for this reason that imaging can be essential to confirming a diagnosis that requires prompt management. Given the clinical urgency of incarceration or strangulation of abdominal hernias, CT imaging is considered superior for both its accuracy in diagnosing the condition as well as its fast turnaround [4]. In contrast, inguinal hernias are better appreciated through magnetic resonance imaging (MRI) rather than CT or ultrasound [5].

In a Richter’s hernia, the portion of the bowel that is herniated may have subsequent compromise of the vasculature, leading to infarction and potential peritonitis secondary to perforation [6]. This type of hernia typically occurs at the femoral ring, inguinal ring, or at sites of previous incisions. The incidence of Richter’s hernias has increased as minimally invasive surgeries have become more popular. Such types of hernias may occur at the site of trocar incisions in laparoscopic procedures, particularly when a 10 millimeter or larger port is used [1,7].

The symptoms of a Richter’s hernia include nausea, vomiting, and crampy abdominal pain. Symptoms are often subclinical with consequent late presentation. CT imaging is typically diagnostic, as in this case. It is instrumental to repair Richter’s hernias as they are at higher risk of progression to gangrene in comparison to other hernia types, consequently with increased risk of mortality. Repair typically involves a preperitoneal approach with laparotomy and potential bowel resection, in addition to repair of the fascial defect [2,8]. The distal ileum is the most commonly involved portion of the intestine, though any aspect of the intestine may become incarcerated [1]. There are few reports describing involvement of the cecum in a Richter’s hernia [2,9].

Our patient is a female who presents with a history of multiple abdominal or pelvic surgeries, increasing the index of suspicion for a hernia as the source of her prolonged abdominal discomfort on presentation to the clinic. The insidious nature and late presentation of a Richter’s hernia may lead to late diagnosis or misdiagnosis [1,2]. Evaluation of a Richter’s hernia is therefore promptly warranted in a patient with a high index of suspicion. It is worth noting that Richter’s hernias are more common in females [1].

The operative management of a Richter’s hernia depends on bowel viability. With viable bowel, no bowel resection would be needed, but ischemic bowel may require a resection in addition to repair of the defect in the fascia [1]. If a Richter’s hernia is reducible, it may be treated in an outpatient setting as an elective procedure. Given the high likelihood of progression to strangulation and necrosis, strangulated Richter’s hernias should be managed urgently; immediate operative exploration is imperative. Traditionally, an open surgical procedure is preferred in cases of strangulation. Although an open approach is traditionally performed, a minimally invasive approach can be considered because it can allow for more extensive exploration of the abdomen. In addition, a minimally invasive approach can cause less injury to intraperitoneal organs and the abdominal wall. It is recommended to repair large defects with prosthetic mesh. However, the use of mesh placement in cases of strangulated Richter’s hernias remains controversial as a portion of bowel may be resected, and therefore the use of mesh in this setting is contingent upon a clinician’s judgement [1,7].

## Conclusions

The patient did not report any concerns regarding this case at the time of writing this report. Despite its rarity, a Richter’s hernia is a more dangerous type of hernia with high risk of strangulation and necrosis. The authors urge clinicians to be mindful of this rare type of hernia for its high risk of mortality in patients.
